# A Neonatal Model of Intravenous *Staphylococcus epidermidis* Infection in Mice <24 h Old Enables Characterization of Early Innate Immune Responses

**DOI:** 10.1371/journal.pone.0043897

**Published:** 2012-09-06

**Authors:** Kenny D. Kronforst, Christy J. Mancuso, Matthew Pettengill, Jana Ninkovic, Melanie R. Power Coombs, Chad Stevens, Michael Otto, Carina Mallard, Xiaoyang Wang, Donald Goldmann, Ofer Levy

**Affiliations:** 1 Newborn Medicine, Boston Children's Hospital, Boston, Massachusetts, United States of America; 2 Infectious Diseases, Boston Children's Hospital, Boston, Massachusetts, United States of America; 3 Harvard Medical School, Boston, Massachusetts, United States of America; 4 Pathology, Dalhousie University, Halifax, Nova Scotia, Canada; 5 Pathogen Molecular Genetics Section, Laboratory of Human Bacterial Pathogenesis, National Institute of Allergy and Infectious Diseases, National Institute of Health, Bethesda, Maryland, United States of America; 6 Physiology, The University of Gothenburg, Gothenburg, Sweden; Columbia University, United States of America

## Abstract

*Staphylococcus epidermidis* (SE) causes late onset sepsis and significant morbidity in catheterized preterm newborns. Animal models of SE infection are useful in characterizing disease mechanisms and are an important approach to developing improved diagnostics and therapeutics. Current murine models of neonatal bacterial infection employ intraperitoneal or subcutaneous routes at several days of age, and may, therefore, not accurately reflect distinct features of innate immune responses to bacteremia. In this study we developed, validated, and characterized a murine model of intravenous (IV) infection in neonatal mice <24 hours (h) old to describe the early innate immune response to SE. C57BL/6 mice <24 h old were injected IV with 10^6^, 10^7^, 10^8^ colony-forming units (CFU) of SE 1457, a clinical isolate from a central catheter infection. A prospective injection scoring system was developed and validated, with only high quality injections analyzed. Newborn mice were euthanized between 2 and 48 h post-injection and spleen, liver, and blood collected to assess bacterial viability, gene expression, and cytokine production. High quality IV injections demonstrated inoculum-dependent infection of spleen, liver and blood. Within 2 h of injection, SE induced selective transcription of TLR2 and MyD88 in the liver, and increased systemic production of plasma IL-6 and TNF-α. Despite clearance of bacteremia and solid organ infection within 48 h, inoculum-dependent impairment in weight gain was noted. We conclude that a model of IV SE infection in neonatal mice <24 h old is feasible, demonstrating inoculum-dependent infection of solid organs and a pattern of bacteremia, rapid and selective innate immune activation, and impairment of weight gain typical of infected human neonates. This novel model can now be used to characterize immune ontogeny, evaluate infection biomarkers, and assess preventative and therapeutic modalities.

## Introduction

Human neonates express a distinct, but incompletely characterized, immune response to infection. This response is heavily dependent on innate immunity due to early qualitative and quantitative deficiencies in the adaptive immune system [1], [2]. Innate immune function is itself dependent on both gestational and postnatal age, and is essential for host defense against infection [2], [3], [4]. Accordingly, preterm newborns are particularly susceptible to invasive bacteria. In addition to this age-dependent activity, neonatal immune responses rely heavily on the expression of pattern recognition receptors such as *Toll*-like receptors (TLRs) [2], [5], [6]. Notably, there is a specific pattern of TLR-mediated cytokine production by neonatal mononuclear cells, monocytes and antigen-presenting cells that is skewed toward low production of pro-inflammatory Th1-polarizing cytokines (e.g., TNF-α) and high production of cytokines with anti-inflammatory or Th2-polarizing activities (e.g., IL-6) [2], [3], [7], [8], [9], [10], [11]. A number of recent studies have shed light on the distinct responses of newborns to TLR agonists *in vitro* [12], [13], but much remains to be learned regarding interactions of the newborn with live bacterial pathogens *in vivo*.

*Staphylococcus epidermidis* (SE) is the most common cause of late onset sepsis in neonatal intensive care units worldwide and disproportionately affects preterm newborns [14], [15], [16], [17], [18]. SE expresses multiple virulence factors, including those that enhance biofilm formation and resist phagocytic killing [19]. SE also releases soluble factors that activate host cells via TLR2 [19], [20], [21]. Although SE infection results in relatively low case mortality (<2%), it is associated with significant long-term morbidity, especially among infants <30 weeks gestation, prolonging length of hospitalization and significantly increasing health care costs [14], [15], [22]. Recent epidemiologic data suggest that sepsis due to Gram-positive bacteria may be associated with neurodevelopmental impairment and poor long-term neurodevelopmental outcomes [15], [23], [24], [25], [26]. Therefore, there is an unmet medical need to prevent and enhance treatment of SE infection [27]. In this context, preclinical animal infection models are a key approach to characterize host-pathogen interactions and inform development of novel biomarkers and anti-infective agents [28]. Several newborn mouse models of staphylococcal infection have been described. However, these have largely focused on mice 2 to 7 days of age and employed either intra-peritoneal or subcutaneous routes of infection [29], [30], [31], [32], [33], [34], [35]. Additionally, while lymphoid organ development and immunity in mice at 7 days is comparable to term human neonates, there is little information regarding the ontogeny of the mouse immune system early enough to compare to human preterms [3], [36]. Because age and route of infection can play fundamental roles in both risk of infection and immune responses to SE [37], [38], we sought to develop a model in newborn mice <24 h old via the IV route.

Herein, we report a novel approach to characterize neonatal host-pathogen interactions by establishing a neonatal model of IV SE infection in mice <24 h old that demonstrates both inoculum-dependent infection of blood and solid organs and activation of innate immune responses. Using this novel model, we demonstrate that in response to SE bacteremia, newborn mice rapidly and selectively up-regulate transcription of TLR2 and MyD88, and mount a systemic cytokine response. Although these early innate immune responses are associated with clearance of SE infection, this host-pathogen interaction results in impairment of weight gain, an important marker of neonatal well-being [30], [39], [40].

## Methods

### Ethics Statement

All animal protocols were approved by the Animal Care and Use Committee of Boston Children's Hospital (11-11-2076R).

### Bacteria

Our studies utilized SE 1457, a clinical strain from an adult patient with a central catheter infection that was previously isolated by Mack and colleagues [41]. Of note, SE 1457 expresses multiple virulence factors, including biofilm forming compounds (poly-γ-glutamic acid), polysaccharide intercellular adhesins, and phenol soluble modulins [19], [42].

### Preparation of Inoculum

500 µl of bacterial stock were added to 30 ml trypticase soy broth (TSB) in a 125 ml baffled flask. Bacteria were grown for 16–20 h in an incubator/shaker at 37°C and 240 RPM. Following overnight incubation, a 1∶20 dilution of the starter stock was made in a 5 ml TSB tube and optical density (OD) was measured at 600 nm. The requisite volume of bacteria, based upon an SE density factor of 0.68×10^9^ (SE/ml) per OD, was harvested by centrifugation at 3000×G for 5 minutes at 4°C. The pellet was resuspended in 1 ml of sterile, pyrogen-free saline (Baxter Healthcare Corp, Deerfield, IL). Serial dilutions in saline yielded bacterial concentrations of 2×10^7^, 2×10^8^, and 2×10^9^ SE/ml, such that a 50 µl dose contained 10^6^, 10^7^, 10^8^ SE.

### Mice

C57BL/6 mice were obtained from Jackson Laboratory (Bar Harbor, ME) and housed in the animal research facilities at Boston Children's Hospital. All procedures were in accordance with an IACUC–approved animal protocol.

### Bacteremia Model

**Figure 1:**
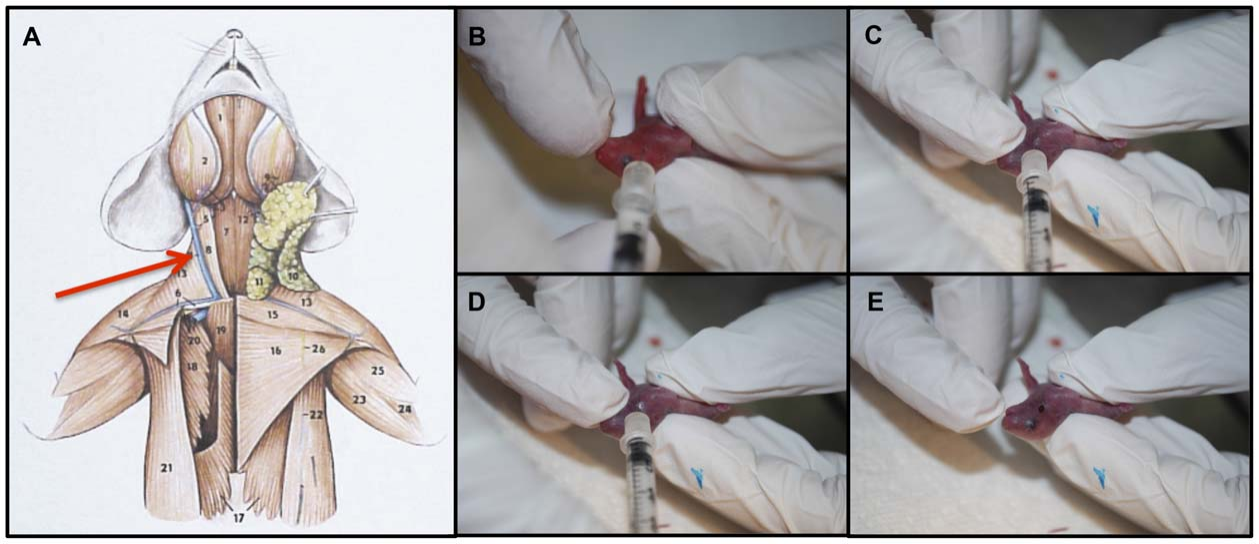
Intrajugular injection of SE in mice less than 24 h old. A) Depiction of murine anatomy including the ventral muscles and blood vessels of the neck, highlighting the location of the external jugular vein (image used with permission of Nature Publishing Group). B) Positioning of mouse and exposure of neck for injection into right external jugular vein. C) Insertion of needle into external jugular vein. D) Injection of inoculum into external jugular vein. E) Removal of needle showing blood return following vessel puncture.

**Figure 2:**
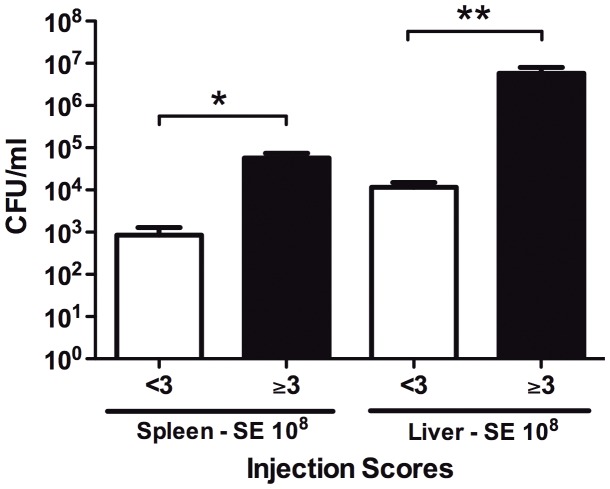
Induction of bacteremia is proportional to injection scoring criteria. Newborn mice less than 24 h old were injected with 10^8^ CFU of SE. Mice were euthanized, organs harvested, and CFUs measured. CFU data were stratified by injection score, determined as per Table 2. Prospective injection scores of 3 or higher correlated with substantially greater CFUs in mouse spleen and liver (N = 5–8, ** p<0.01, *** p<0.001, Mann-Whitney t-test).

Mouse pups <24 h old were used for all experiments. Gravid dams were monitored on a daily basis during the expected week of parturition to confirm age of pups. Additionally, age was verified using pup appearance (newborns were pink with translucent skin) [43] and weight at the time of the experiment. Pups were injected via the intra-jugular route with 50 µl of either saline or 10^6^, 10^7^, or 10^8^ CFUs of SE. Dose range was selected based on experiments demonstrating systemic cytokine responses (e.g., IL-6) at those doses. This range was also consistent with bacterial inocula reported in other murine models [30], [44]. Injections utilized a two-person technique adapted from Kienstra et al (Table 1, Video S1) [45]. One investigator restrained each pup by pinning their right forelimb to their body with thumb and extending their neck with index finger to expose the external jugular vein on the right side (Figure 1A, B). A second investigator then approached the external jugular vein at a 10–20 degree angle (Figure 1C), inserted needle and delivered the inoculum (Figure 1D). Following injection, the first investigator provided pressure for hemostasis at the site of injection (Figure 1E), monitored for signs of distress, and marked pups for future identification. Both investigators then determined an injection score based on a previously validated scoring scale (Table 2). Only injections with scores of 3 or greater were included in subsequent analyses (Figure 2). After several months of training, investigators routinely achieved high quality injections (scores of 3–5) on ∼70–80% of pups. Following injections, mice were returned to their cage and left for a predetermined time interval of 2, 24, or 48 h. At the indicated time-points, pups were weighed and sacrificed via decapitation or terminal cardiac puncture for organ extraction, bacterial culture and cytokine profiling.

**Table 1:** Technique diagram for intra-jugular injection of newborn mice.

Step	Description
1	Person 1 holds pup and monitors for signs of distress
2	Person 1 pushes mouse right forelimb down with thumb and uses index finger to pull head back in order to extend neck and expose the external jugular vein
3	Person 2 approaches the external jugular at a 10–20 degree angle and inserts needle bevel down
4	Person 2 then administers injection of 50 ul of inoculum at desired concentration (slowly) and pulls out
5	Person 1 provides gentle pressure for hemostasis, marks pup for identification and replaces pup in cage
6	Person 1 and 2 determine injection score based on scoring table

**Table 2:** Injection scoring criteria.

Score	Definition
1	Extravasation of inoculum into soft tissue (no blood seen on needle extraction)
2	Knicking of the vessel but inoculum mostly in the soft tissue (mostly inoculum seen on needle extraction)
3	Inoculum injected mostly into the vessel with some bubbling of surrounding soft tissue (blood and inoculum seen on needle extraction)
4	Inoculum injected mostly into the vessel with minimal bubbling of surrounding soft tissue (blood and inoculum seen on needle extraction)
5	Inoculum injected within the vessel (blood seen on extraction of needle with no soft tissue swelling)

### Bacterial Recovery from Murine Organs and Immunologic Response to Infection

To ensure maximal sterility, work surfaces and instruments were cleaned with 70% ethanol and RNAse Zap (Applied Biosystems/Ambion, Austin, TX). At a predetermined time-point, mice were either sacrificed via decapitation or cardiac puncture. Cardiac puncture was performed following the technique outlined by Hoff et al [46]. 20–30 µl of blood were collected and placed in 5 µl pyrogen-free heparin sodium (1,000 USP units/ml; Sagent Pharmaceuticals, Schaumburg, IL) on ice for cytokine analyses and quantification of bacterial growth. To expose the abdominal contents, a transverse incision was made through the skin at the level of the umbilicus. Similarly, a vertical incision was made starting at the umbilicus toward the diaphragm. Spleen and liver were identified in the left and right upper quadrants of the abdominal cavity, respectively. The spleen was dissected away from the stomach with forceps and placed into a 1.7 ml microcentrifuge tube containing 2.5 mm glass beads (Biospec Products, Inc, Bartlesville, OK) up to the 1 ml line and 780 µl of saline. The multi-lobed liver was removed by clipping its fascial connection to the diaphragm and then dividing into two equal halves. One half was prepared for culture in the same manner as the spleen while the other was placed in one volume of RNAlater (QIAGEN, Valencia, CA) for gene expression studies. Tubes containing spleen and liver tissue were homogenized using a Mini-Beadbeater-16 (Biospec Products, Inc) for 1 minute. Tissue homogenates were then prepared for plating at mulitple dilutions (spleen at neat, 1∶10, and 1∶100; liver at 1∶10, 1∶100, and 1∶1000) to ensure CFUs were accurately counted. Dilutions were made with sterile saline. 50 µl samples were plated onto Trypticase soy agar (TSA) with 5% sheep blood plates (BD Diagnostics, Franklin Lakes, NJ) and placed in an incubator at 37°C with 5% CO_2_ for 16–24 h. Liver halves placed in RNAlater solution were stored at −20°C. Mice were weighed pre-injection and immediately prior to decapitation/cardiac puncture to assess percent weight change following SE challenge.

### Purification of mouse liver RNA for gene expression analysis

All pipettes/surfaces were cleaned with RNase Zap to prevent degradation of RNA. Liver RNA samples were preserved in RNAlater and stored at −20°C. RNA was isolated using the RNeasy Mini Kit and RNeasy MinElute Cleanup Kit, per the manufacturer's instructions (QIAGEN). RNA concentration and purity were measured using a Nanodrop 1000 spectrophotometer (Thermo Fisher Scientific, DE).

### Quantitative real time PCR (qRT-PCR) for gene expression analysis

Expression levels of selected genes were assessed by qRT-PCR analysis using an ABI 7300 real-time PCR system and software (Applied Biosystems, Foster City, CA). mRNA (100 ng) was reverse-transcribed to cDNA using the RT^2^ First Strand Kit (QIAGEN) according to the manufacturer's instructions. The equivalent of approximately 1 ng RNA/well was assayed using the RT^2^ Profiler PCR Array System according to the manufacturer's instructions. We utilized a Mouse *Toll*-like Receptor Signaling PCR Array (PAMM-018, QIAGEN) containing primers for 84 genes of interest and 12 controls. Controls included 5 housekeeping genes (glucuronidase beta, hypoxanthine guanine phosphoribosyl transferase, heat shock protein 90 alpha [cytosolic] class B member 1, glyceraldehyde-3-phosphate dehydrogenase, and actin beta), mouse genomic DNA contamination-, 2 reverse transcription-, and 2 positive PCR controls. mRNA levels were normalized to housekeeping genes and quantified using the ΔΔ comparative threshold (Ct) method using the analysis tools provided by QIAGEN (http://www.sabiosciences.com/pcr/arrayanalysis.php).

### Cytokine Quantification

Plasma IL-6 and TNF-α were determined by ELISA as per the manufacturer's instructions (eBioscience, San Diego, CA, USA). Additionally, a panel of mouse cytokine and chemokines (TNF-α, MCP-1, IL-6, KC, IL-12 (p70), G-CSF, GM-CSF, IL-10, IL-1β, and IPL-10) were measured in diluted heparinized plasma, using the Milliplex Map Mouse Cytokine/Chemokine 10-Plex Immunoassay Kit (Millipore, Chicago, IL). Data were acquired on a Milliplex Analyzer Luminex 100 machine and analyzed using xPonent 3.1 software (Millipore) according to the manufacturer's instructions.

### Genetic analyses

Samples of mouse tail tissue were genotyped for XX vs. XY using real time PCR by Transnetyx (Cordova, TN).

### Statistical Analyses

Statistical analyses of mRNA expression employed commercially available on-line software (QIAGEN) using a Student's t-test of the replicate 2^−ΔCt^ values for each gene in the control and test groups. Graphpad Prism 5.0a Software (San Diego, CA) was used to perform all statistical analyses. Unless otherwise stated, all data were based on experiments with injection scores ≥3. Data groups with unequal variances were analyzed using a Mann-Whitney unpaired t-test. A lower limit of detection of 50 CFU/ml was determined and used for statistical analyses. P-values<0.05 were considered significant.

## Results

### Feasibility of a newborn IV SE infection model

We developed a novel model of IV SE infection in newborn mice less than 24 h old, incorporating techniques developed by Kienstra et al describing intravascular injection of fluorescent dextran into newborn mice [45]. Accordingly, we injected 50 µl of SE 1457 into the external jugular vein of each animal (Figure 1, and Video S1). Each injection was scored and animals were categorized by injections into acceptable (scores 3–5) and unacceptable (scores 1 or 2) groups. To test the validity of the scoring system, we euthanized animals at 2 h and harvested organs to compare bacterial growth in spleen and liver homogenates. Animals with injections scores in the acceptable group demonstrated approximately 2–3 logs greater CFUs in spleen and liver, validating our scoring system (Figure 2).

### Inoculum-dependent infection of blood and solid organs

**Figure 3:**
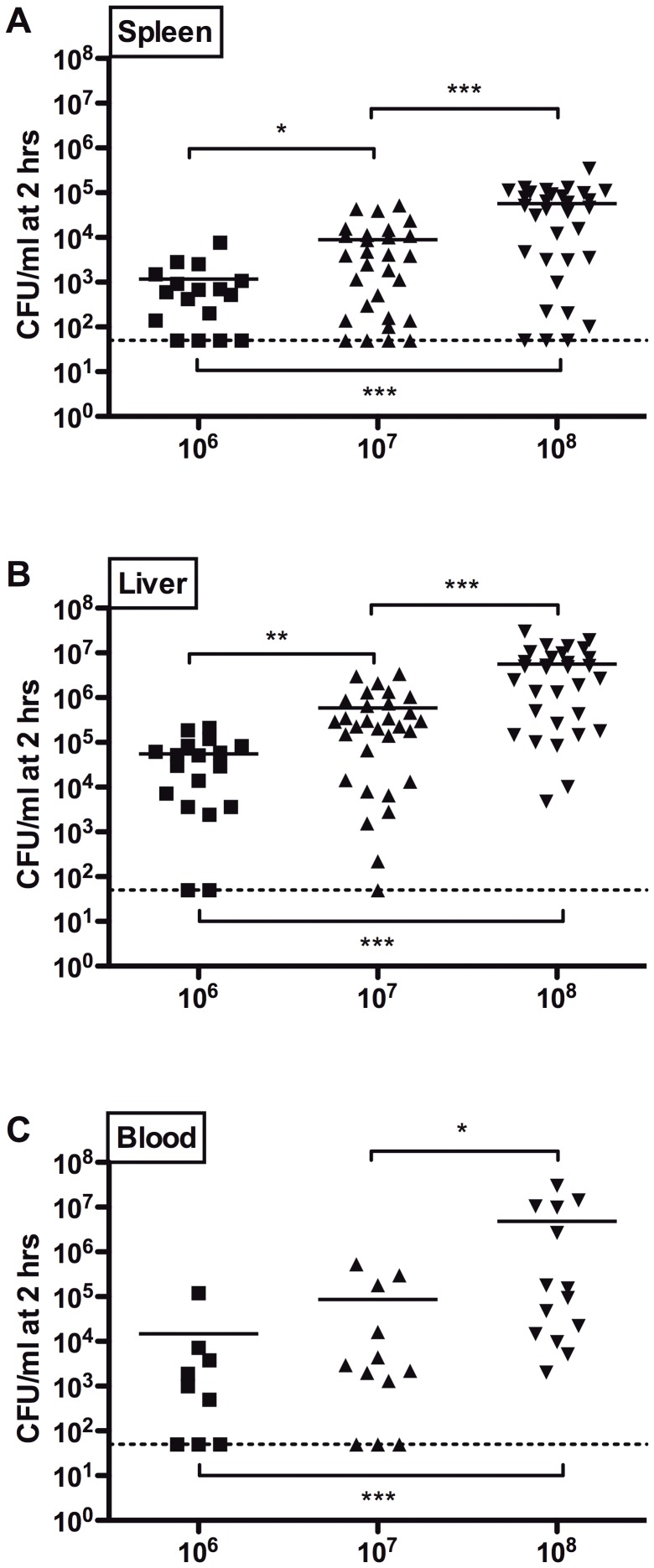
SE-induced inoculum-dependent infection of solid organs and blood. Bacterial load in A) spleen, B) liver, and C) blood at 2 h following intra-jugular injection of SE at 10^6^, 10^7^, and 10^8^ CFU showing inoculum-dependent increase in bacterial counts. Graph represents individual data points with median values indicated by a horizontal line. Only mice with injection scores of 3–5 were used in analysis. Groups were compared using the Mann-Whitney t-test (N = 7–31, * p<0.05, ** p<0.01, *** p<0.001).

We next determined inoculum effects of SE infection on spleen, liver and blood, comparing inocula of 10^6^, 10^7^ and 10^8^ CFU at 2 h post-injection (Figure 3). Only acceptable injection scores were included in these analyses. We noted inoculum-dependent infection, as indicated by greater CFUs, in each of these anatomic compartments (Figure 3). No differences in mean CFUs were noted when male and female newborns were compared (Figure S1).

### Clearance of SE bacteremia by neonatal mice within 24–48 h of infection

**Figure 4:**
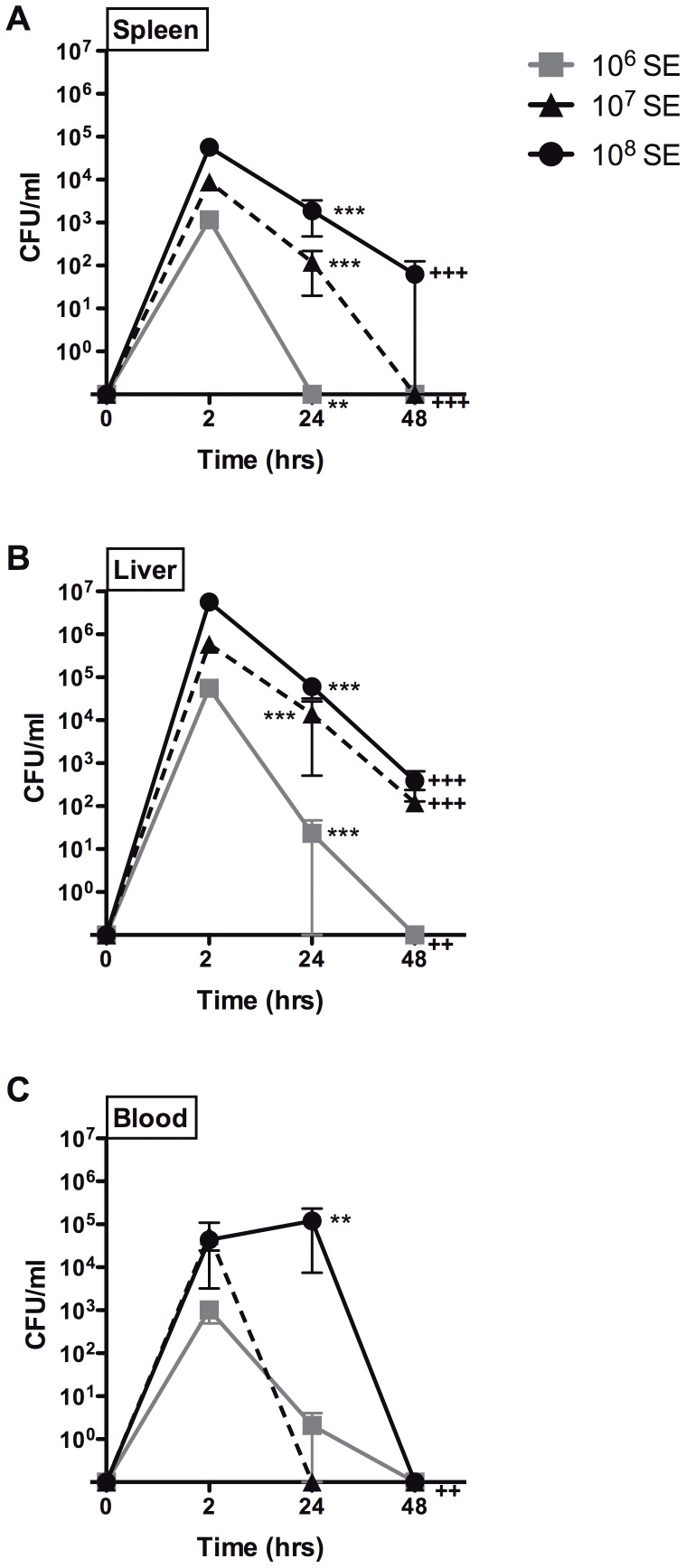
Neonatal mice clear SE infection within 48 h post-injection. Effect of time on bacterial load in A) spleen, B) liver, and C) blood following intra-jugular injection of SE at 10^6^, 10^7^, and 10^8^ CFU shows significant clearance of bacteria by 48 h when compared to inoculation at 2 h (N = 5–31, ** p_24 h_<0.01, *** p_24 h_<0.001, ++p_48 h_<0.01, +++p_48 h_<0.001, Mann-Whitney t-test).

To assess the course of SE infection in newborn mice, we measured bacterial CFUs in spleen, liver and blood at 2, 24 and 48 h post-injection. Overall, there was a significant decrease in the number of CFUs demonstrated by all organs and across all inocula from 2 h to 48 h (p<0.001) (Figure 4). This suggests a natural ability of newborn mice to gradually clear SE infection. Of note, no mortality was observed during this 48 h post-injection period.

### SE infection triggers activation of a TLR transcriptome in liver

**Figure 5:**
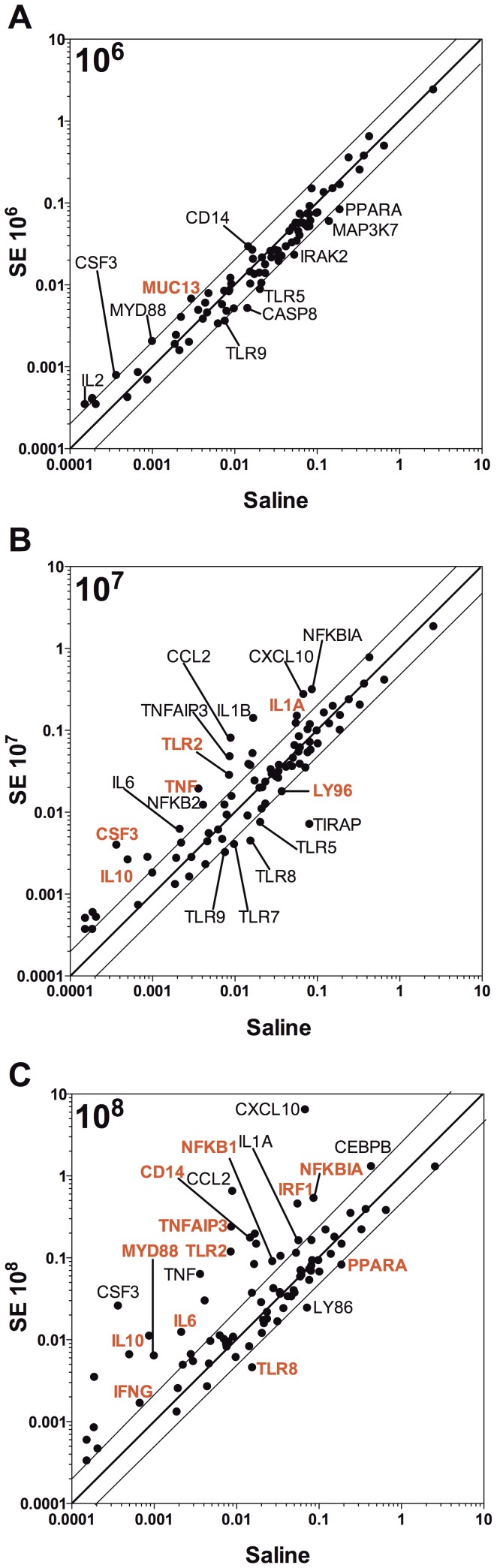
SE inoculum-dependent transcription of TLR signaling pathway genes. Neonatal mice were injected IV with SE at A) 10^6^, B) 10^7^, and C) 10^8^ CFU and euthanized at 2 h. Liver tissue was harvested, RNA isolated and transcription of TLR-signaling pathway genes assessed. Log_10_ normalized gene expression levels are shown for both saline (x-axis) and inoculum SE (y-axis) injected mice. Inoculum-dependent up-regulation of TLR-gene transcription is evident, with mRNA transcripts that are significantly up- or down-regulated shown in red.

**Figure 6:**
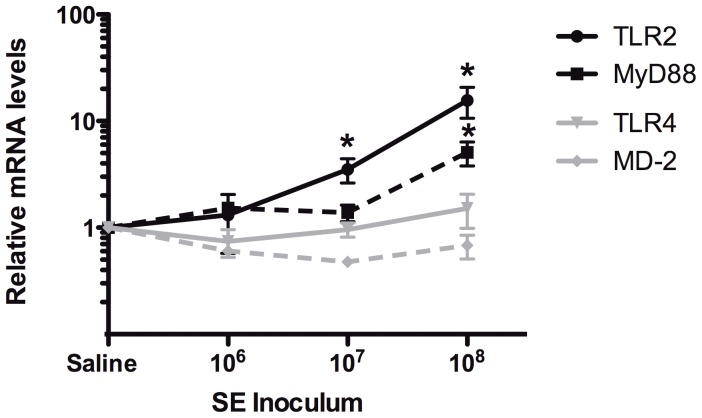
SE induces selective mRNA expression of TLR2 and MyD88. Liver samples were collected 2 h post-SE injection and analyzed for mRNA expression of TLR2, TLR4, MyD88, and MD-2. Relative fold change in mRNA transcripts, each normalized to housekeeping genes as described in methods, was expressed as a ratio of each transcript in the SE injected animals to that in saline-injected animals. SE induced significant inoculum-dependent increases in mRNAs encoding TLR2 and MyD88 (* p<0.05, Student t-test). By contrast, TLR4 and MD-2 were not up-regulated over saline-injected mice.

To assess the impact of SE infection on transcription of innate immunity-related genes, we harvested livers 2 h post SE injection, isolated RNA and analyzed it by qRT-PCR using TLR signaling pathway gene arrays (Table S1). SE induced inoculum-dependent increases in mRNA transcripts encoding multiple innate immune genes, cytokines, and chemokines (Figure 5 and Table 3). Among the transcripts significantly increased after injection of 10^8^ CFU were pattern-recognition receptors (TLR2, CD14), adaptor molecules (MyD88, FADD), transcription factors (IP_3_, IRF-1, Jun, NFkB1, CEBP), and cytokines (TNF-α, IL1β, IL10, and CSF-2 [GM-CSF]). When data were analyzed as a function of inocula, SE injection with 10^7^ and 10^8^ CFU resulted in the selective up-regulation of TLR2 (p<0.05, Figure 6), but not any of the other eight TLRs measured. Likewise, CD14 mRNA was significantly increased after SE injection (p<0.05, Figure 5). MyD88, a key TLR adaptor molecule, was also up-regulated in response to the 10^8^ inoculum (p<0.05, Figure 6).

**Table 3:** Changes in liver mRNA regulation 2 h following injection with 10^8^ SE relative to control.

Up Regulation		P Value	No Change		P Value	No Change		P Value	Down Regulation		P Value
Cxcl10	96.32	0.073	Muc13	1.86	0.105	Tradd	−1.34	0.315	**Ppara**	**−2.25**	*0.040
Ccl2	74.48	0.080	Il1r1	1.85	0.311	Nfrkb	−1.35	0.355	Ly86	−2.9	0.114
Csf3	72.03	0.143	Cd80	1.82	0.186	Irak1	−1.36	0.488	**Tlr8**	**−3.33**	*0.009
**Tnfaip3**	**28.04**	*0.027	Btk	1.53	0.373	Hspa1a	−1.4	0.621			
Ptgs2	18.93	0.136	Pglyrp1	1.47	0.232	Nr2c2	−1.41	0.105			
Tnf	17.65	0.114	Ticam2	1.46	0.307	Chuk	−1.47	0.504			
**Tlr2**	**14.06**	*0.044	Tlr4	1.45	0.425	Il6ra	−1.48	0.372			
**Il10**	**13.36**	*0.000	Irf3	1.33	0.785	Ly96	−1.52	0.245			
Clec4e	12.93	0.144	Nfkbil1	1.2	0.664	Tlr7	−1.58	0.216			
**Cd14**	**12.17**	*0.028	Ticam1	1.2	0.959	Tlr6	−1.61	0.470			
**Il1b**	**11.87**	*0.020	Tlr1	1.2	0.779	Tlr5	−1.67	0.286			
Ripk2	8.61	0.121	Hras1	1.19	0.378	Hmgb1	−1.69	0.365			
**Irf1**	**8.44**	*0.026	Tbk1	1.19	0.431	Casp8	−1.71	0.648			
Nfkb2	7.38	0.125	Mapk9	1.14	0.701	Mapk8	−1.86	0.121			
**Myd88**	**6.5**	*0.039	Eif2ak2	1.14	0.528	Hspd1	−1.96	0.259			
**Nfkbia**	**6.32**	*0.036	Tnfrsf1a	1.12	0.612						
Il6	5.87	0.122	Elk1	1.11	0.759						
**Jun**	**5.15**	*0.010	Tlr9	1.09	0.688						
**Csf2**	**4.62**	*0.049	Traf6	1.08	0.952						
Ifnb1	3.98	0.139	Ube2v1	1.07	0.644						
**Nfkb1**	**3.38**	*0.020	Cd86	1.02	0.744						
Rel	3.12	0.051	Map2k4	1.01	0.804						
**Cebpb**	**3.09**	*0.018	Tollip	1	0.953						
Il1a	2.92	0.080	Peli1	−1.04	0.970						
Ifng	2.55	0.125	Agfg1	−1.05	0.956						
Fos	2.45	0.357	Ikbkb	−1.06	0.702						
**Fadd**	**2.41**	*0.049	Tirap	−1.14	0.766						
Lta	2.3	0.179	Tlr3	−1.2	0.987						
Nfkbib	2.24	0.200	Map3k1	−1.22	0.587						
Il2	2.23	0.190	Mapk8ip3	−1.22	0.582						
Irak2	2.2	0.153	Map3k7	−1.24	0.394						
Rela	2.04	0.057	Map2k3	−1.26	0.566						
Il12a	2.02	0.161	Ube2n	−1.29	0.890						

### SE infection triggers a high ratio of systemic IL-6/TNF-α production

**Figure 7:**
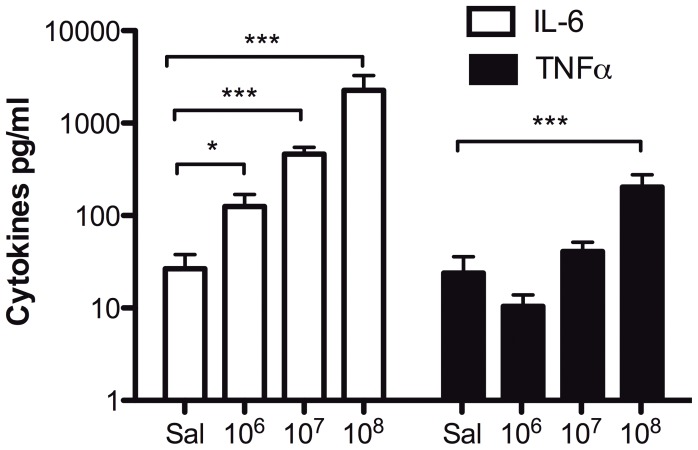
SE-induced systemic IL-6 and TNF-α production. IL-6 and TNF-α were both up-regulated in response to intra-jugular injection with SE showing a high ratio of IL-6 to TNF-α at 2 h post-injection. IL-6 was induced in an inoculum-dependent manner following injection with 10^6^, 10^7^ and 10^8^ CFUs. TNF-α was also induced, but at substantially lower concentrations, with significance only at the highest inoculum when compared to saline injections (N = 4–9, * p<0.05, ** p<0.01, *** p<0.001, Mann-Whitney t-test).

**Figure 8:**
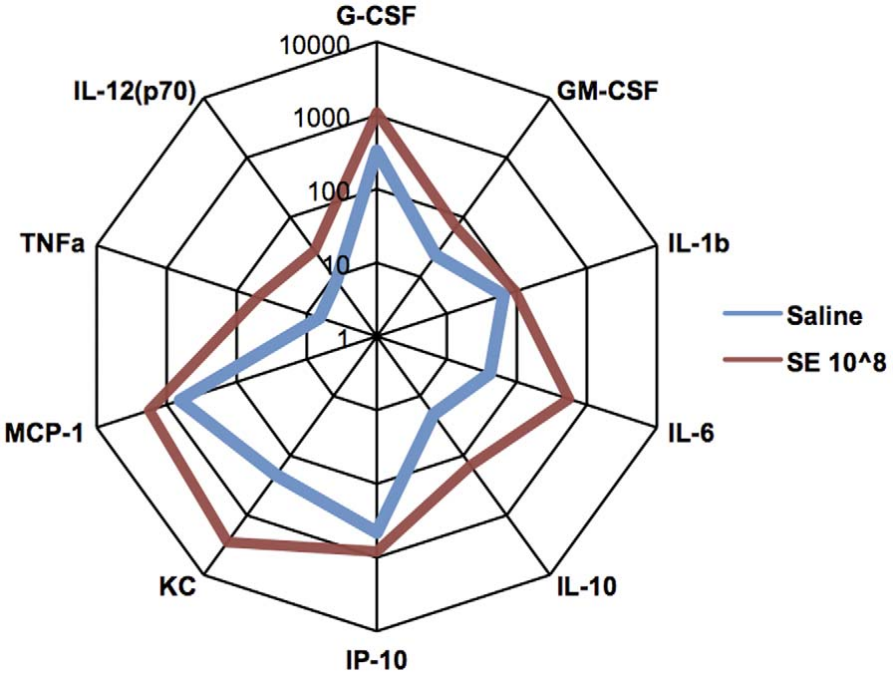
SE induced systemic cytokine production. Radar plot representation of Milliplex cytokine data that highlights the fold change in cytokine induction demonstrating up-regulation of both IL-6 and TNF-α in mice injected with 10^8^ CFU of SE at 2 h. Results represent 3 independent experiments.

To confirm protein level expression of key cytokines and to assess the relation of our model to known patterns of human neonatal cytokine expression, we measured concentrations of IL-6 and TNF-α in plasma by ELISA (Figure 7). SE induced inoculum-dependent production of both cytokines with IL-6 being induced with greater potency and magnitude than TNF-α, a pattern typically seen in human newborns with Gram-positive bacteremia [2]. Additionally, multiplex cytokine analysis suggested SE-induced production of multiple additional cytokines including the neutrophil chemoattractant KC (CXCL1), colony-stimulating factors GM-CSF (CSF-2) and G-CSF (CSF-3), and the anti-inflammatory cytokine IL-10 (Figure 8).

### SE infection results in inoculum-dependent impairment in neonatal weight gain

**Figure 9:**
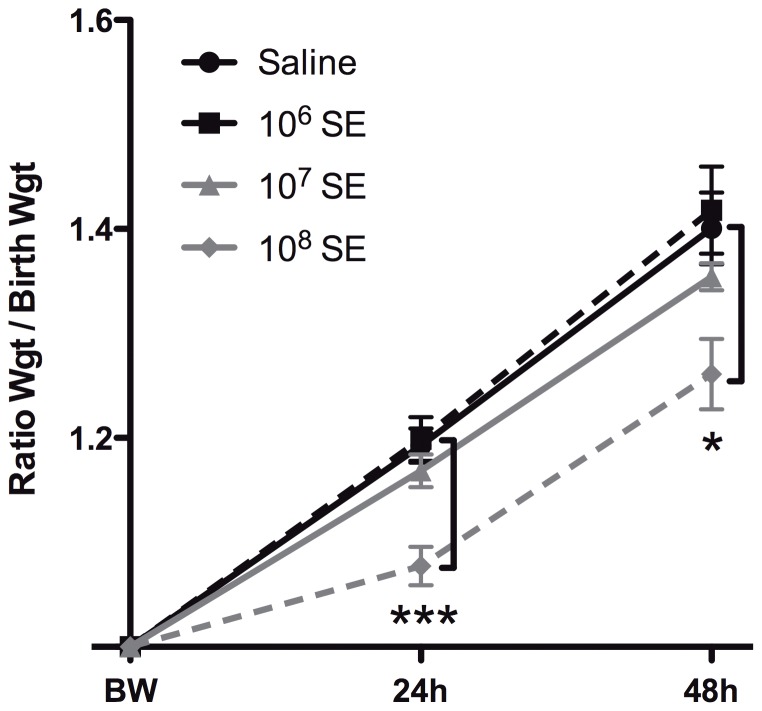
SE inoculum-dependent impairment of neonatal weight gain. Newborn pups were weighed at birth (birth weight = BW) and at 24 and 48 h following injection with saline or the indicated inocula of SE. There was significant impairment of weight gain noted in mice injected with 10^8^ CFU of SE. This pattern of growth was observed at both 24 and 48 h (N = 5–13, * p<0.05, *** p<0.001, Mann-Whitney t-test).

Though newborn mice rapidly clear SE infection, exposure to SE can trigger systemic inflammation with potentially pathologic effects. We therefore measured weight as a sensitive marker of neonatal well-being following SE injection. When normalized to each animal's birth weight, weight gain at both 24 and 48 h was significantly impaired in animals injected with 10^8^ CFU (p_24 h_<0.001, p_48 h_<0.05, Figure 9).

## Discussion

Our study has established, for the first time, a neonatal model of IV SE infection in mice less than 24 h old. This model uses a clinically relevant age group and route of infection to study early neonatal host-pathogen interactions and mechanisms of host defense to SE. A prospectively validated injection scoring system permits its use as a standard operating procedure. Our model demonstrates inoculum-dependent infection of solid organs and blood, activation of innate immune responses, bacterial clearance by 24 to 48 h post-infection, and impairment of neonatal weight gain. These features make this neonatal mouse model a useful tool to study host and pathogen determinants of infection.

There are several parallels between mice and humans that contribute to our model's utility for evaluating neonatal SE infection. Similar to human newborns who are able to clear SE bacteremia within days, neonatal mice cleared SE from blood and organs within 24 to 48 h of injection, demonstrating a natural ability to overcome infection [47]. Clearance of SE bacteremia was also associated with early activation of innate immune responses. In particular, SE induced inoculum-dependent mRNA transcription in the liver with increased expression of important cytokines such as IL-6, an acute phase reactant known to be up-regulated in infected human newborns [2], [7], [8], [47]. SE-injected mice also demonstrated selective increases in liver mRNA encoding TLR2, a key innate immune receptor that mediates recognition and clearance of multiple Gram-positive organisms including SE [20], [48], [49], [50], [51], and of the TLR adaptor molecule, MyD88 [52]. Interestingly, transcription of TLR2 and MyD88 are both coordinately up-regulated during human neonatal Gram-positive infection *in vivo* [53], [54], highlighting how our murine model recapitulates the human neonatal response to Gram-positive bacterial infection. SE also induced inoculum-dependent impairment in weight gain, a sensitive marker of neonatal well-being known to be negatively affected by infection in both mice and human newborns [39], [40]. Overall, these similarities suggest that our model captures key characteristics of neonatal host-bacterial interaction, highlighting its potential value.

A fundamental aspect of our model is its ability to demonstrate the impact of inflammation following SE infection. Specifically, impairment of weight gain likely reflects SE-induced inflammation, including systemic cytokine/chemokine induction. Our model provides a venue to study additional effects of SE bacteremia on neonatal physiology, including potential effects on perinatal brain development and neurodevelopmental outcomes, an area of increased biomedical focus [23], [24], [25], [26], [55], [56].

In conclusion, we have established and prospectively validated a neonatal model of IV SE infection in mice less than 24 h old. This model demonstrates inoculum-dependent blood and solid organ infection associated with innate immune activation as indicated by selective induction of TLR-signaling pathway genes and corresponding systemic cytokine production. Though the systemic cytokine response is associated with bacterial clearance, it also likely contributes to impairment of weight gain reflecting downstream effects of inflammation. Long-term evaluation and treatment of septic neonates will need to focus on balancing inflammation and bacterial killing. To this end, our model will enable mechanistic studies of host-pathogen interactions and development of novel diagnostics and therapeutics.

## Supporting Information

Figure S1 **Comparison of bacterial burden in solid organs of female and male mice.** Neonatal pups were injected with 10^8^ CFU of SE and euthanized at 2 h for harvest of spleen and liver. Organ homogenates were plated for bacterial CFU. Pup tissue was genotyped using real time PCR. Mean CFUs were similar for female and male pups (N = 3–7, Mann-Whitney t-test).
(PDF)

Video S1 **Intra-jugular injection in newborn mice.** Video illustrates the necessary steps for performing accurate intra-jugular injection in newborn mice (injection sore 4). These steps are further described in table 1.
(M4V)

Table S1 **Summary of gene products used in the mouse TLR signaling array.**
(PDF)
