# Supplementary figures and images for: A Neonatal Model of Intravenous Staphylococcus epidermidis Infection in Mice <24 h Old Enables Characterization of Early Innate Immune Responses
Source: PLoS One. 2012 Sep 6;7(9):e43897. doi: 10.1371/journal.pone.0043897 (PMC3435332; doi:10.1371/journal.pone.0043897)

Figure S1.

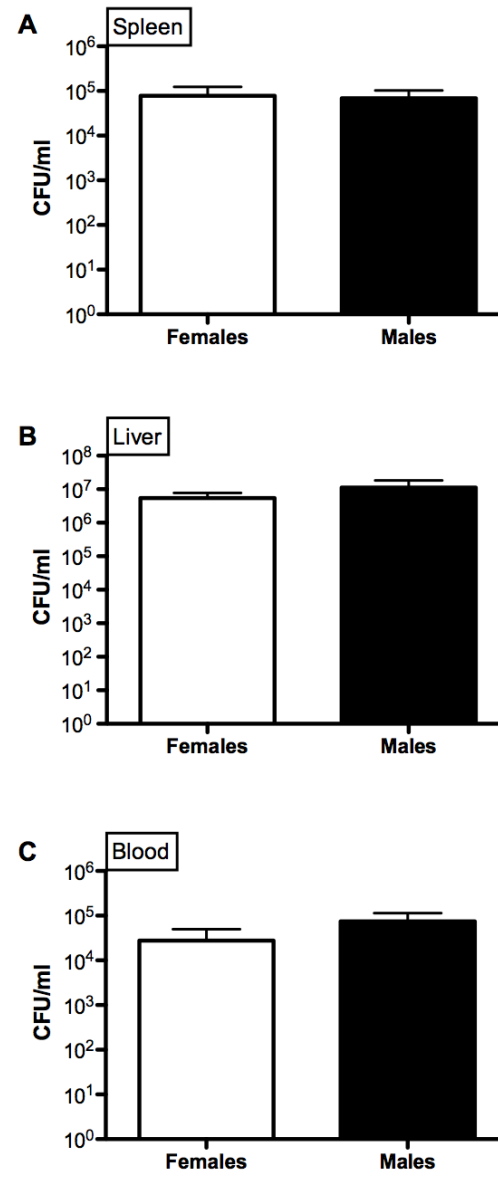

Supplement: Figure S1 — Comparison of bacterial burden in solid organs of female and male mice. Neonatal pups were injected with 108 CFU of SE and euthanized at 2 h for harvest of spleen and liver. Organ homogenates were plated for bacterial CFU. Pup tissue was genotyped using real time PCR. Mean CFUs were similar for female and male pups (N = 3–7, Mann-Whitney t-test). (PDF) [file pone.0043897.s001.pdf]
