# Supplementary material for: A Neonatal Model of Intravenous Staphylococcus epidermidis Infection in Mice <24 h Old Enables Characterization of Early Innate Immune Responses
Source: PLoS One. 2012 Sep 6;7(9):e43897. doi: 10.1371/journal.pone.0043897 (PMC3435332; doi:10.1371/journal.pone.0043897)
Supplement: Table S1 — Summary of gene products used in the mouse TLR signaling array. (PDF) [file pone.0043897.s003.pdf]

Table S1. Summary of gene products used in the mouse TLR signaling array

Symbol	GenBank	Description	Symbol	GenBank	Description
Btk	NM_013482	Bruton agammaglobulinemia tyrosine kinase	Map3k1	NM_011945	Mitogen-activated protein kinase kinase kinase 1
Casp8	NM_009812	Caspase 8	Map3k7	NM_172688	Mitogen-activated protein kinase kinase kinase 7
Ccl2	NM_011333	Chemokine (C-C motif) ligand 2	Mapk8	NM_016700	Mitogen-activated protein kinase 8
Cd14	NM_009841	CD14 antigen	Mapk8ip3	NM_013931	Mitogen-activated protein kinase 8 interacting protein 3
Cd80	NM_009855	CD80 antigen	Mapk9	NM_016961	Mitogen-activated protein kinase 9
Cd86	NM_019388	CD86 antigen	Myd88	NM_010851	Myeloid differentiation primary response gene 88
Cebpb	NM_009883	CCAAT/enhancer binding protein (C/EBP), beta	Nfkb1	NM_008689	Nuclear factor of kappa light polypeptide gene enhancer in B-cells 1, p105
Chuk	NM_007700	Conserved helix-loop-helix ubiquitous kinase	Nfkb2	NM_019408	Nuclear factor of kappa light polypeptide gene enhancer in B-cells 2, p49/p100
Clec4e	NM_019948	C-type lectin domain family 4, member e	Nfkbia	NM_010907	Nuclear factor of kappa light polypeptide gene enhancer in B-cells inhibitor, alpha
Csf2	NM_009969	Colony stimulating factor 2 (granulocyte-macrophage)	Nfkbib	NM_010908	Nuclear factor of kappa light polypeptide gene enhancer in B-cells inhibitor, beta
Csf3	NM_009971	Colony stimulating factor 3 (granulocyte)	Nfkbil1	NM_010909	Nuclear factor of kappa light polypeptide gene enhancer in B-cells inhibitor-like 1
Cxcl10	NM_021274	Chemokine (C-X-C motif) ligand 10	Nfrkb	NM_172766	Nuclear factor related to kappa B binding protein
Elk1	NM_007922	ELK1, member of ETS oncogene family	Nr2c2	NM_011630	Nuclear receptor subfamily 2, group C, member 2
Fadd	NM_010175	Fas (TNFRSF6)-associated via death domain	Peli1	NM_023324	Pellino 1
Fos	NM_010234	FBJ osteosarcoma oncogene	Pglyrp1	NM_009402	Peptidoglycan recognition protein 1
Hmgb1	NM_010439	High mobility group box 1	Ppara	NM_011144	Peroxisome proliferator activated receptor alpha
Hras1	NM_008284	Harvey rat sarcoma virus oncogene 1	Eif2ak2	NM_011163	Eukaryotic translation initiation factor 2-alpha kinase 2
Agfg1	NM_010472	ArfGAP with FG repeats 1	Ptgs2	NM_011198	Prostaglandin-endoperoxide synthase 2
Hspa1a	NM_010479	Heat shock protein 1A	Rel	NM_009044	Reticuloendotheliosis oncogene
Hspd1	NM_010477	Heat shock protein 1 (chaperonin)	Rela	NM_009045	V-rel reticuloendotheliosis viral oncogene homolog A (avian)
Ifnb1	NM_010510	Interferon beta 1, fibroblast	Ripk2	NM_138952	Receptor (TNFRSF)-interacting serine-threonine kinase 2

Ifng	NM_008337	Interferon gamma	Tbk1	NM_019786	TANK-binding kinase 1
Ikbkb	NM_010546	Inhibitor of kappaB kinase beta	Ticam1	NM_174989	Toll-like receptor adaptor molecule 1
Il10	NM_010548	Interleukin 10	Ticam2	NM_173394	Toll-like receptor adaptor molecule 2
Il12a	NM_008351	Interleukin 12A	Tirap	NM_054096	Toll-interleukin 1 receptor (TIR) domain-containing adaptor protein
Il1a	NM_010554	Interleukin 1 alpha	Tlr1	NM_030682	Toll-like receptor 1
Il1b	NM_008361	Interleukin 1 beta	Tlr2	NM_011905	Toll-like receptor 2
Il1r1	NM_008362	Interleukin 1 receptor, type I	Tlr3	NM_126166	Toll-like receptor 3
Il2	NM_008366	Interleukin 2	Tlr4	NM_021297	Toll-like receptor 4
Il6	NM_031168	Interleukin 6	Tlr5	NM_016928	Toll-like receptor 5
Il6ra	NM_010559	Interleukin 6 receptor, alpha	Tlr6	NM_011604	Toll-like receptor 6
Irak1	NM_008363	Interleukin-1 receptor-associated kinase 1	Tlr7	NM_133211	Toll-like receptor 7
Irak2	NM_172161	Interleukin-1 receptor-associated kinase 2	Tlr8	NM_133212	Toll-like receptor 8
Irf1	NM_008390	Interferon regulatory factor 1	Tlr9	NM_031178	Toll-like receptor 9
Irf3	NM_016849	Interferon regulatory factor 3	Tnf	NM_013693	Tumor necrosis factor
Jun	NM_010591	Jun oncogene	Tnfaip3	NM_009397	Tumor necrosis factor, alpha-induced protein 3
Lta	NM_010735	Lymphotoxin A	Tnfrsf1a	NM_011609	Tumor necrosis factor receptor superfamily, member 1a
Muc13	NM_010739	Mucin 13, epithelial transmembrane	Tollip	NM_023764	Toll interacting protein
Ly86	NM_010745	Lymphocyte antigen 86	Tradd	NM_0010331 61	TNFRSF1A-associated via death domain
Ly96	NM_016923	Lymphocyte antigen 96	Traf6	NM_009424	Tnf receptor-associated factor 6
Map2k3	NM_008928	Mitogen-activated protein kinase kinase 3	Ube2n	NM_080560	Ubiquitin-conjugating enzyme E2N
Map2k4	NM_009157	Mitogen-activated protein kinase kinase 4	Ube2v1	NM_023230	Ubiquitin-conjugating enzyme E2 variant 1
